# Comparing Quality of Public Primary Care between Hong Kong and Shanghai Using Validated Patient Assessment Tools

**DOI:** 10.1371/journal.pone.0121269

**Published:** 2015-03-31

**Authors:** Xiaolin Wei, Haitao Li, Nan Yang, Samuel Y. S. Wong, Onikepe Owolabi, Jianguang Xu, Leiyu Shi, Jinling Tang, Donald Li, Sian M. Griffiths

**Affiliations:** 1 School of Public Health, Prince of Wales Hospital, Shatin, NT, Hong Kong SAR, China; 2 Faculty of Epidemiology and Population Health, London School of Hygiene and Tropical Medicine, Keppel Street, WC1E 7HT, London, United Kingdom; 3 Shanghai Commission of Health and Family Planning, 300 Shibo County Road, Shanghai, 200125, China; 4 Hampton House, 615 N Wolfe St #5041, Baltimore, Maryland 21205, United States of America; 5 Admiralty Centre Tower 1, 18 Harcourt Road, Hong Kong, Hong Kong SAR, China; Old Dominion University, UNITED STATES

## Abstract

**Objectives:**

Primary care is the key element of health reform in China. The objective of this study was to compare patient assessed quality of public primary care between Hong Kong, a city with established primary care environment influenced by its colonial history, and Shanghai, a city leading primary care reform in Mainland China; and to measure the equity of care in the two cities.

**Methods:**

Cross sectional stratified random sampling surveys were conducted in 2011. Data were collected from 1,994 respondents in Hong Kong and 811 respondents in Shanghai. A validated Chinese version of the primary care assessment tool was employed to assess perceived quality of primary care with respect to socioeconomic characteristics and health status.

**Results:**

We analyzed 391 and 725 respondents in Hong Kong and Shanghai, respectively, who were regular public primary care users. Respondents in Hong Kong reported significant lower scores in first contact accessibility (1.59 vs. 2.15), continuity of care (2.33 vs. 3.10), coordination of information (2.84 vs. 3.64), comprehensiveness service availability (2.43 vs. 3.31), comprehensiveness service provided (2.11 vs. 2.40), and the total score (23.40 vs. 27.40), but higher scores in first contact utilization (3.15 vs. 2.54) and coordination of services (2.67 vs. 2.40) when compared with those in Shanghai. Respondents with higher income reported a significantly higher total primary care score in Hong Kong, but not in Shanghai.

**Conclusions:**

Respondents in Shanghai reported better quality of public primary care than those in Hong Kong, while quality of public primary care tended to be more equitable in Shanghai.

## Introduction

Primary care responds to common health problems that account for the vast majority of the population’s health needs. Studies have demonstrated that better primary care is associated with improved health outcomes and reduced health inequity [1–4]. Countries in different regions of the world are moving towards primary care oriented health systems based on the WHO call in 2008 [5]. However, equitable distribution of high quality primary care remains an ultimate but out of reach goal in many countries [6].

Urban areas have an increasing need for primary care because of their large populations and relatively high income inequality distribution [7]. Hong Kong and Shanghai are among the largest metropolitan cities in the world. They have similar health indicators while both face the challenge of a rapidly growing aging population. Hong Kong, as a former British colony and a current special administrative region in China, retains its former socioeconomic policies where the government funds most of inpatient services, while the majority (over 70%) of primary care is provided through private doctors [8]. Government outpatient clinics (GOPCs) provide about 20% of primary care services. Most GOPCs open during office hours with some on evenings and Saturdays. GOPCs are operated with a fixed quota of patient consultations on each day. Patients need to make an appointment prior to a consultation. For any visit to a specialist in public hospitals except for emergencies, patients need to obtain referral from the GOPC or a private doctor. Though opening to the general public, GOPCs targeted for patients with chronic illnesses, those who are poor and civil servants. GOPCs is heavily subsidized by the government and only charge a flat visit fee of HK$45 (US$ 6) which is waived for those who are on government social assistance. GOPCs provide acute care and limited preventive services.

In mainland China, the over-dependence on hospitals for minor illnesses has made its healthcare less accessible and more expensive to the public [9]. Since 2009, China has implemented a comprehensive health reform plan which strengthened the roles of primary care facilities, i.e., community health centers (CHC) in the urban areas and township hospitals in the rural areas [10]. All CHCs in Shanghai are fully funded by the government with a mandate to provide integrated health services including acute care, preventive care, health education and promotion, rehabilitation, chronic disease management and technical support for family planning to a population of around 50,000 within the catchment area of a CHC. CHCs are walk-in clinics, opening in regular office hours with some in evenings and on weekends. The average cost of a consultation is 20 RMB (US$ 3.25) in 2011 and most can be covered by health insurance.[11] In Shanghai, patients may seek care directly in big hospitals without being referred from primary care doctors. CHCs in Shanghai provide service to the general public, but specifically address health needs of the elderly, the poor and those with chronic diseases.

Comparing the quality of primary care in different health systems can provide insights into the impact of primary care policies. However, most current studies only measured primary care within one health system. This study aims to compare patient assessments of the quality of public primary care in Hong Kong and Shanghai, and to measure the equity of care. It focuses on public primary care providers because they play a critical role in reaching the population with the greatest needs and are intended to reduce health inequity across the population. Users of public primary care in both cities are relatively poor, have chronic diseases and are usually the elderly [12, 13]. Since Hong Kong and Shanghai represent the top-tier in healthcare delivery in China, this study could shed light on the current state-of-the-art in primary care delivery in China and identify strengths and weaknesses in specific domains of primary care that the rest of the country could use as benchmarks for comparison.

## Methods

### Ethical consideration

Ethical approval was obtained from the Ethics Committees of the Chinese University of Hong Kong and New Territories East Cluster Clinical Research (Ref. No. CRE-2010.441). We have obtained written consents from patients in Shanghai where a face-to-face survey was conducted. In Hong Kong, due to the nature of telephone survey, written consents were not available. The surveyors have adequately informed the participants of the study purpose and their rights to stop at any time. Oral consents were obtained and recorded for those who participated in the survey.

### Survey design and procedures

We conducted cross-sectional stratified random surveys in Hong Kong and Shanghai. The minimum sample size in each city was calculated as 368 with a 95% confidence interval and power of 80% [14], based on an estimated difference of 1.2 PCAT score with a standard deviation of 5.3 [15]. In Hong Kong, we applied population based telephone surveys to identify GOPC users. In Shanghai, we conducted clusters based surveys to identify CHC users. Thus, a design effect of 2 was applied to double the sample size in Shanghai in order to offset the cluster effect [16]. Therefore, we aimed to collect data from 368 respondents in Hong Kong and 736 respondents in Shanghai who regarded the public primary care facilities as their usual source of care, which is defined as the provider that the respondents usually visit when they are sick or need health advice [17].

Because only 20% of patients in Hong Kong use public primary care, we planned to conduct 2,000 telephone interviews to achieve a sample size of 368 GOPC users. A stratified random telephone survey was conducted with Chinese-speaking residents of Hong Kong aged 18 and above in October and November 2011. Three major geographic regions (north, middle and south) were stratified and 16,662 telephone numbers were randomly selected from a telephone directory. No interviews were attempted at non-Chinese households (about 1% in Hong Kong), commercial numbers, or fax numbers. The household member whose past birthday was the closest to the day of interview was invited to join the study. At least four telephone calls were made at different time periods before considering the number to be invalid. In total, 2,932 valid household contacts were obtained. Among them, 1,994 completed the interview, with a response rate of 68%. Of the 938 participants who did not complete the interview, 912 refused to join, and 26 stopped in the middle.

In Shanghai, we conducted a stratified random survey by face-to-face interviews in November 2011 because a representative residential telephone registry was not available in the city. We firstly divided the city into four geographical areas (north, south, east and west). Under each geographical area, one district was randomly selected. Then one CHC was randomly selected under each district. In total, four CHCs were selected. In each CHC, we systematically sampled every 5^th^ patient aged 18 and above to conduct exit interviews until 200 patients were reached. In the end, a total of 811 respondents completed the survey with a response rate of 94%.

### Primary care assessment tools

We assessed patient experience with primary care providers in the two cities using the Chinese adult version of Primary Care Assessment Tool (PCAT)—Short Edition, a tool developed by the Johns Hopkins Primary Care Center [18, 19], and then translated, piloted, and validated in both Cantonese and Mandarin Chinese [20–22]. The two versions of PCAT questionnaire contain the same questions in the same sequences, with only slight differences of wording due to dialects. The instrument measures key attributes of primary care that are related to effective primary care organization and delivery at the population level [18, 19]. First contact (two domains) is defined as the accessibility to (4 items) and use of primary care services (3 items) when a new health or medical problem arises. First contact utilization reflects the gate-keeping role of primary care providers, while ensuring first contact accessibility prevent patients from becoming sicker [23]. Continuity of care refers to the longitudinal use of a regular source of primary care over time (4 items). For example, enhanced continuity of care is associated with better compliance with medications [24]. Coordination (two domains) refers to the interpersonal linkage of care between different levels of providers (4 items) or informational linkage of care through using the electronic information system (3 items). The integrated information system can assist with access to patients’ medical records, which can further enhance doctors’ abilities to recognize patients’ problems and therapies [25]. Comprehensiveness (two domains) refers to the availability of clinical and preventative services within the provider (4 items), as well as the actual provision of care during consultations (5 items). Family centeredness is defined as the inclusion of family health concerns in decision-making (3 items). Community orientation refers to the provider’s knowledge of community health needs (3 items). Cultural competence is defined as patient’s willingness to recommend the primary care provider to others (2 items) [19]. All questions address the patients’ regular primary care provider with a 4-point Likert-type scale [26]. The total primary care score was calculated by adding the mean scores of each of the ten domains [17].

In addition, basic demographic and socioeconomic information was collected such as gender, age, household income, education, occupation, and health insurance status. We grouped employment status into two categories, those who had a job, including people identified as self-employed, and those who did not have a job, i.e., the retired, housewives and the unemployed. In Shanghai, health insurance referred to government backed Basic Medical Insurance Schemes [27]; while in Hong Kong, it referred to employer-provided or self-purchased health insurance. Income groups were classified according to the city’s poverty line and median household income level in 2011 [28, 29]. Any households with a monthly income below the city poverty line, i.e., RMB 3,000 (US$484) in Shanghai and HK$ 10,000 (US$ 1282) in Hong Kong, were regarded as having low income; while any households having an income above the median level, i.e., RMB 10,000 (US$1,613) in Shanghai and HK$ 25,000 (US$ 3,205) were regarded as having high income.

### Statistical analysis

We employed Chi-square tests to compare the socio-demographic characteristics and health care utilization measures of the respondents from the two cities. Independent two-sample t-tests were firstly employed to compare scores of items, domains and the total score of PCAT between the two cities, while multiple linear regression models were then employed to compare differences of PCAT scores between the two cities by controlling for respondents’ gender, age, education, occupation, household income levels, self-reported health status and presence of chronic diseases. The city effect was reported using adjusted beta with 95% confidence intervals (CIs) where Shanghai was regarded as the reference group. In measuring inequities within each city, we used ANOVA to test the differences among respondents with different household income levels within each city. For all tests conducted in the study, a P-value less than 0.05 was adopted as the statistically significant level. All analyses were conducted using SPSS19.0 (Chicago, IL).

## Results

We analyzed data from the 391 respondents in Hong Kong and 725 respondents in Shanghai who regarded a public primary care provider as their regular source of care. In both cities, the majority of respondents tended to be females, had an education level at middle school or below, did not have a job, and had used their public primary care provider for over five years (Table 1). Nearly all respondents in Shanghai were insured, while only 13% in Hong Kong reported having health insurance. Compared with those in Hong Kong, respondents in Shanghai were more likely to be older, having poorer health status, insured and visiting public clinics more frequently.

**Table 1:** Socioeconomic and demographic characteristics, and health care measures of the public primary care users in Hong Kong and Shanghai.

Characteristics	Hong Kong (%) (n = 391)	Shanghai (%) (n = 725)	P-value^1^
***Demographics***			
Gender			<0.001
Female	219(56.0)	497(68.6)	
Male	172(44.0)	228(31.4)	
Age group			<0.001
≤44	89(22.8)	39(5.4)	
45~59	119(30.4)	214(29.5)	
≥60	183(46.8)	472(65.1)	
Self-reported health status			<0.001
Good and above	198(50.6)	174(24.0)	
Fair	170(43.5)	449(61.9)	
Poor	23(5.9)	102(14.1)	
Having any physical, mental or emotional problem			<0.001
Yes	122(31.2)	600(82.8)	
No	269(68.8)	125(17.2)	
***Socioeconomics***			
Education			0.001
College and above	72(18.4)	151(20.8)	
High school or equivalent	108(27.6)	267(36.8)	
Middle school and below	211(54.0)	307(42.3)	
Occupation			0.008
Have a job	69(17.6)	85(11.7)	
Do not have a job	322(82.4)	640(88.3)	
Household income			<0.001
Below poverty line	99(25.3)	111(15.3)	
Between poverty line and median	114(29.2)	519(71.6)	
Above median	45(11.5)	72(9.9)	
Reject to answer	133(34.0)	23(3.2)	
Health insurance			<0.001
Yes	52(13.3)	697(96.1)	
No	339(86.7)	28(3.9)	
***Visits to the primary care provider***			
Number of visits in the last year, n(%)			<0.001
≤3 visits	170(43.5)	67(9.2)	
4~6 visits	155(39.6)	57(7.9)	
≥7 visits	66(16.9)	601(82.9)	
Length of time with the health facility			<0.001
≤1 year	23(5.9)	57(7.9)	
1~2 years	27(6.9)	92(12.7)	
3~4 years	27(6.9)	94(13.0)	
≥5 years	314(80.3)	482(66.5)	

^1.^ Chi-square test was used to examine the differences between the participants from the two cities using relative number.

**Fig 1:**
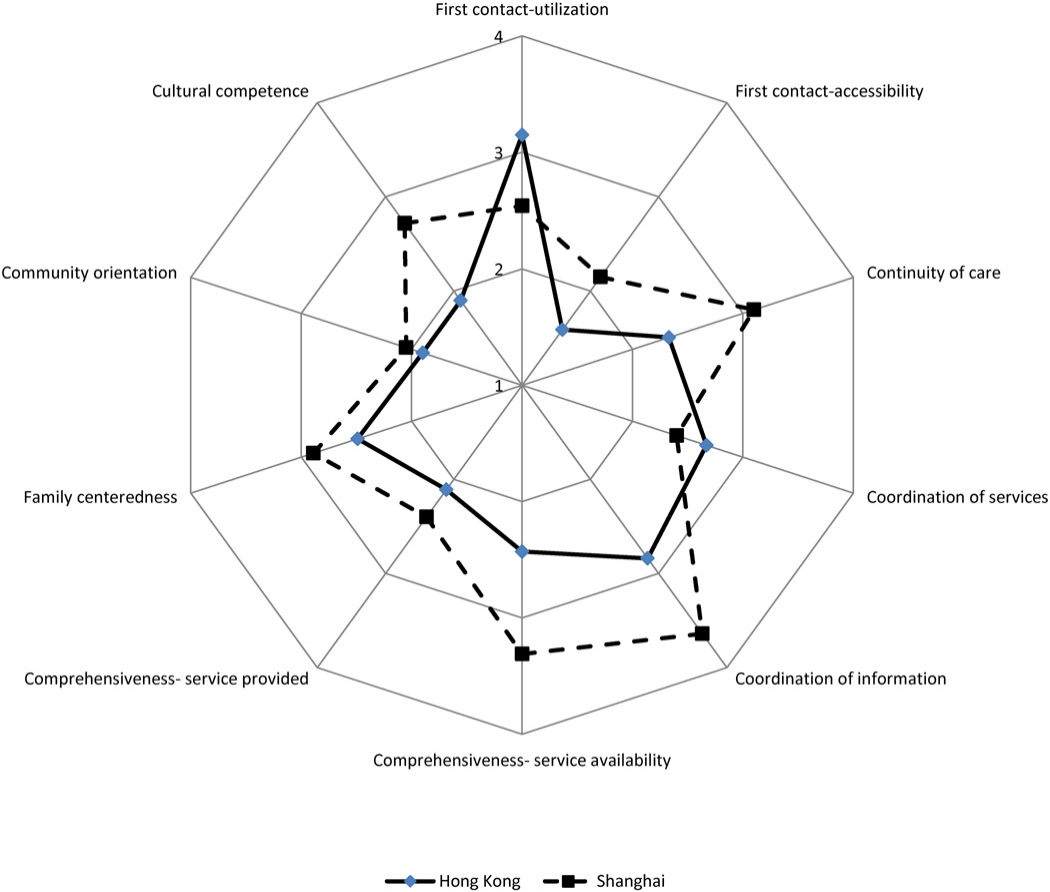
Individual attributes scores of the Primary Care Assessment Tools reported by the respondents in Hong Kong and Shanghai in 2011.

After adjusting for demographic, socio-economic and health factors, respondents in Hong Kong reported higher scores for first contact—utilization (3.15 vs. 2.54, Table 2) and coordination of services (2.67 vs. 2.40), but lower scores for all other domains, including first contact—accessibility (1.59 vs. 2.15), continuity of care (2.33 vs. 3.10), coordination of information (2.84 vs. 3.64), comprehensiveness-service availability (2.43 vs. 3.31), comprehensiveness- service provided (2.11 vs. 2.40), family centeredness, community orientation, cultural competence and total primary care score (23. 40 vs. 27.20) when compared with those in Shanghai (Fig 1). After controlling for confounders, respondents in Shanghai reported approximately 4 points higher for total score than those in Hong Kong. The largest differences between the two cities were observed in comprehensiveness-service availability (1.008), cultural competence (0.889), continuity of care (0.715), and first contact- accessibility (0.635).

**Table 2:** Individual and total primary care attributes scores reported by respondents in Hong Kong and Shanghai.

Attributes	Hong Kong (SD)	Shanghai (SD) ^1^	Adjusted city effect (95%CI)^2^
First contact-utilization	3.15(0.84)	2.54(0.58) ***	0.757(0.647,0.866)^###^
First contact-accessibility	1.59(0.49)	2.15(0.45) ***	-0.635(-0.709, -0.562) ^###^
Continuity of care	2.33(0.60)	3.10(0.58) ***	-0.715(-0.811, -0.620) ^###^
Coordination of services	2.67(0.67)	2.40(0.61) ***	0.225(0.121,0.329) ^###^
Coordination of information	2.84(0.57)	3.64(0.48) ***	-0.704(-0.788, -0.620) ^###^
Comprehensiveness-service availability	2.43(0.75)	3.31(0.50) ***	-1.008(-1.106, -0.910) ^###^
Comprehensiveness-service provided	2.11(0.70)	2.40(0.61) ***	-0.296(-0.402, -0.191) ^###^
Familycenteredness	2.49(0.80)	2.89(0.84) ***	-0.466(-0.600, -0.332) ^###^
Communityorientation	1.90(0.60)	2.05(0.73) ***	-0.166(-0.279, -0.053)^##^
Cultural competence	1.90(0.75)	2.72(1.13) ***	-0.889(-1.055, -0.723) ^###^
Totalscore	23.40(3.18)	27.20(3.72) ***	-3.898(-4.479, -3.317) ^###^

^1^. Compared with that of Hong Kong using independent two-sample t-test

* p<0.05

** p<0.01

*** p<0.001

^2^. The city effect was examined using multiple linear regression models, where the dependent variable is the domain score, while independent variables are gender, age, education, occupation, household income, health status and presence of chronic diseases

^#^ p<0.05

^##^ p<0.01

^###^ p<0.001;

β is calculated with Shanghai as reference; CI = confidence interval.

For first contact-utilization, respondents in Hong Kong (2.82) were more likely to choose their primary care providers as their first choice for health check-up compared with those in Shanghai (2.42). When seeing a specialist, respondents in Hong Kong (3.34) were more likely to seek the approval from the primary care provider than those in Shanghai (1.65). Respondents in Shanghai (3.69) reported a higher score regarding access to the public primary care providers than those in Hong Kong (2.55), because they were more likely to see the doctor in the same day. Regarding continuity of care, respondents in Shanghai were far more likely to be seen by the same doctor (2.91) and to be known as a person rather than a patient (2.58) compared with GOPC users in Hong Kong (1.96 and 1.86, respectively). In terms of coordination of information, more respondents in Shanghai (3.97) reported having brought their medical records from the primary care provider to a specialist when compared with those in Hong Kong (2.04). Compared with GOPCs in Hong Kong, CHCs in Shanghai provide more acute and public health care, including sewing up a wound (3.71 vs. 2.46) and mental health counseling (2.82 vs. 2.01). In terms of service provision, more respondents in Shanghai reported receiving advices from doctors than those in Hong Kong on healthy dietary (3.04 vs. 2.44), exercise (2.84 vs. 2.33) and medications (3.22 vs. 2.53). Public primary care doctors in Hong Kong were less likely to consult patient or his/her family’s ideas when planning the treatment (2.31 vs. 3.39). Patients in Shanghai (2.64) were more likely to receive traditional Chinese medicine service than those in Hong Kong (1.79). Significant differences (P<0.001) were identified on each item after adjusting for patient characteristics. (Table 3)

**Table 3:** Mean scores of the selected items under each domain reported by respondents in Hong Kong and Shanghai.

Items	Hong Kong(SD)	Shanghai(SD)^1^	Adjusted city effect (95%CI)^2^
**First contact—utilization**			
B1. When you need a regular general checkup, do you go to your PCP before going somewhere else?	2.82(1.11)	2.42(1.30)***	0.400(0.197,0.603)^###^
B3. When you have to see a specialist, does your PCP have to approve or give you a referral?	3.34(0.94)	1.65(0.48) ***	1.974(1.875,2.073) ^###^
**First contact—accessibility**			
C3. When your PCP is open and you get sick, would someone from there see you the same day?	2.55(0.99)	3.69(0.46) ***	-1.056(-1.158, -0.954) ^###^
C5. When your PCP is closed, is there a phone number you can call when you get sick?	1.21(0.54)	1.69(0.87) ***	-0.629(-0.755, -0.503) ^###^
**Continuity of care**			
D1. When you go to your PCP’s, are you taken care of by the same doctor or nurse each time?	1.96(0.97)	2.91(0.93) ***	-0.805(-0.959, -0.651) ^###^
D7. Does your PCP know you very well as a person, rather than as someone with a medical problem?	1.86(1.02)	2.58(1.01) ***	-0.826(-0.992, -0.660) ^###^
**Coordination of services**			
E10. Did your PCP write down any information for the specialist about the reason for the visit?	2.86(0.83)	2.48(0.78) ***	0.379(0.248,0.510) ^###^
**Coordination of information**			
F1. When you go to your PCP, do you bring any of your own medical records, such as shot records or reports of medical care you had in the past?	2.04(1.08)	3.97(0.25) ***	-1.792(-1.901, -1.684) ^###^
**Comprehensiveness—service availability**			
G8. Counseling for mental health problems	2.01(0.98)	2.82(0.73) ***	-1.018(-1.153, -0.883) ^###^
G10. Sewing up a cut that needs stitches	2.46(1.12)	3.71(0.66) ***	-1.352(-1.491, -1.212) ^###^
**Comprehensiveness—service provided**			
H1. Advice about healthy foods and unhealthy foods or getting enough sleep	2.44(1.12)	3.04(1.06) ***	-0.556(-0.733, -0.380) ^###^
H5. Advice about appropriate exercise for you	2.33(1.13)	2.84(1.08) ***	-0.403(-0.581, -0.225) ^###^
H7. Checking on and discussing the medications you are taking	2.53(1.07)	3.22(0.97) ***	-0.576(-0.741, -0.411) ^###^
**Family centeredness**			
I1. Does your PCP ask you about your ideas and opinions when planning treatment and care for you or a family member?	2.31(1.06)	3.39(0.89) ***	-1.131(-1.287, -0.975) ^###^
**Community orientation**			
J1. Does anyone at your PCP’s office ever make home visits?	1.16(0.53)	1.50(0.86) ***	-0.410(-0.535, -0.285) ^###^
**Cultural competence**			
K3. Would you recommend your PCP to someone who uses folk medicine, such as herbs or homemade medicines, or has special beliefs about health care?	1.79(0.86)	2.64(1.23) ***	-0.981(-1.164, -0.798) ^###^

^1^. Compared with that of Hong Kong using independent two-sample t-test

* p<0.05

** p<0.01

*** p<0.001

^2^. The city effect was examined using multiple linear regression models, where the dependent variable is the item score, while independent variables are gender, age, education, occupation, household income, health status and presence of chronic diseases;

^#^ p<0.05

^##^ p<0.01

^###^ p<0.001

β is calculated with Shanghai as reference; CI = confidence interval

We compared primary care scores between those with middle or high levels of household income and those with low household income within each city (Table 4). In Hong Kong, respondents with high income levels reported a significantly higher total primary care score (24.32). Similarly, respondents with high or middle levels of income in Hong Kong tended to report higher scores for coordination, family centeredness and community orientation. In Shanghai, the high income group reported higher scores for family centeredness but lower scores for first contact utilization when compared with the low income group. No significant differences were found in other domains among different income groups in Shanghai. Higher income levels were significantly associated with higher quality of care in Hong Kong but not in Shanghai (Table 5).

**Table 4:** Individual and total primary care attributes scores (SD, standard deviations) reported by respondents with different household income levels in Hong Kong and Shanghai.

Attributes	Hong Kong			Shanghai		
	Low	Middle	High	Low	Middle	High
First contact-utilization	3.20(0.76)	3.23(0.78)	3.27(0.74)	2.68(0.55)	2.54(0.57)*	2.40(0.61)**
First contact-accessibility	1.56(0.42)	1.52(0.46)	1.62(0.48)	2.19(0.45)	2.12(0.44)	2.30(0.48)
Continuity of care	2.37(0.58)	2.29(0.56)	2.43(0.62)	3.02(0.58)	3.13(0.57)	3.11(0.55)
Coordination of services	2.76(0.70)	2.57(0.71) *	2.76(0.66)	2.45(0.53)	2.37(0.63)	2.41(0.63)
Coordination of information	2.84(0.53)	2.88(0.53)	3.07(0.57) *	3.61(0.55)	3.63(0.47)	3.74(0.40)
Comprehensiveness- service availability	2.34(0.79)	2.39(0.81)	2.44(0.73)	3.26(0.57)	3.32(0.50)	3.34(0.41)
Comprehensiveness-service provided	2.06(0.66)	2.14(0.68)	2.10(0.63)	2.44(0.62)	2.40(0.61)	2.40(0.56)
Familycenteredness	2.18(0.81)	**2.62(0.75)**	**2.69(0.64)**	2.78(0.84)	2.90(0.84)	3.08(0.82) *
Communityorientation	1.80(0.67)	2.00(0.56) *	1.96(0.53)	2.07(0.72)	2.04(0.75)	2.14(0.62)
Cultural competence	1.90(0.76)	1.88(0.76)	1.98(0.76)	2.68(1.00)	2.69(1.16)	2.93(1.02)
Totalscore	23.02(3.36)	23.52(2.86)	24.32(3.16) *	27.19(3.67)	27.14(3.76)	27.84(3.30)

ANOVA tests were conducted for each city. Respondents with low income were used as reference group. Statistically significant results were bolded.

*p<0.05

**p<0.01

***p<0.001.

**Table 5:** Multiple linear regression analysis results of total primary care score in each primary care setting, Shanghai and Hong Kong.

Independent variables	Total primary care score, β(95% CI)	
	Hong Kong (n = 391)	Shanghai (n = 725)
***Demographics***		
Gender		
Female	ref^#^	ref
Male	-0.206(-0.861, 0.449)	-0.054(-0.656, 0.547)
Age group		
≤44	ref	ref
45~59	0.455(-0.504, 1.414)	-2.293(-3.820, -0.767)**
≥60	0.488(-0.524, 1.500)	-2.261(-3.843, -0.679) **
Self reported health status		
Good and above	ref	ref
Fair	-0.258(-1.006, 0.490)	0.172(-0.520, 0.865)
Poor	-0.513(-1.963, 0.938)	-0.220(-1.174, 0.733)
Having any physical, mental or emotional problem		
Yes	ref	ref
No	0.031(-0.715, 0.777)	0.624(-0.235, 1.484)
***Socioeconomics***		
Education		
Middle school and below	ref	ref
High school or equivalent	-0.543(-1.382, 0.295)	-0.252(-0.870, 0.366)
College and above	-0.252(-1.233, 0.730)	-0.943(-1.734, -0.151)*
Occupation		
Have a job	ref	ref
Do not have a job	-0.212(-1.141, 0.717)	-0.288(-1.250, 0.674)
Household income		
Low	ref	ref
Middle	0.775(-0.156, 1.707)	0.130(-0.633, 0.893)
High	1.831(0.552, 3.110) **	1.026(-0.097, 2.150)
Health insurance		
No	ref	ref
Yes	0.005(-0.970, 0.980)	-0.190(-1.694, 1.313)
***Visits to the primary care provider***		
Number of visits in the last year, n(%)		
≤3 visits	ref	ref
4~6 visits	0.059(-0.701, 0.820)	0.721(-0.593, 2.035)
≥7 visits	1.574(0.582, 2.566) **	1.750(0.725, 2.775) **
Length of time with the health facility		
≤1 year	ref	ref
1~2 years	0.586(-1.221, 2.394)	-0.722(-1.954, 0.510)
3~4 years	0.105(-1.684, 1.894)	-1.168(-2.418, 0.083)
≥5 years	0.076(-1.291, 1.442)	0.663(-0.415, 1.741)

^#^ Ref = reference group;

*p<0.05

**p<0.01

CI = Confidence interval

## Discussion

Public primary care users in Shanghai reported higher perceived quality of care for almost all domains except for first contact utilization and coordination of service when compared with those in Hong Kong. Regarding first contact accessibility, patients in Shanghai reported that they were more likely to see a doctor of the CHC on the same day, but patients in Hong Kong had difficulty in booking into the quota system on the same day when getting sick. In addition, patients in Shanghai reported that they were more likely to see the same doctors over time as patients can select doctors, which is a key feature of better continuity [19]. On the contrary, the appointment system in GOPCs did not allow patients to choose doctors. Regarding comprehensiveness, CHCs in Shanghai provided a list of free preventative care under a package funded by the government on a per capita basis. The package included elderly care, chronic disease management, health education, immunisation, maternal and child care, mental health care, communicable disease prevention and reporting [22]. For example, patients with hypertension and diabetes received at least quarterly follow-up consultations in CHCs [30]. In Hong Kong, GOPCs provided limited preventative services such as flu vaccine and follow-up preventative care for people with hypertension and diabetes. Other preventative services, such as maternal and child health care were provided by separate institutions under the Department of Health. Regarding family centeredness, CHC doctors in Shanghai were more likely to involve patients and their families in medical decision-making. Regarding community orientation and cultural competence, CHCs provided traditional Chinese medicine (TCM) that fits with patient needs [31]. In Hong Kong, although TCM was provided in public health facilities, there was little integration or communication between the GOPCs and public TCM clinics despite that the public’s preference to TCM [32].

Higher scores in first contact utilization were reported in Hong Kong because a referral letter from primary care doctors was needed to visit specialists in public hospitals in Hong Kong. In Shanghai, the referral system was not established and health insurance schemes did not limit patient access to specialists without referrals. As a result, many patients directly visited hospitals. In addition, primary care doctors in China gained limited patient trust [33] as the majority of doctors only received short-term in-service training in primary care, but not the three years residency training in family medicine as in countries such as Canada or UK. In the absence of motivating factors and policies, changing de facto health seeking behavior amongst patients seeking specialist care is extremely challenging. Service coordination was better reported in Hong Kong because GOPCs and public hospitals were both under the management of the Hospital Authority. In Shanghai, CHCs and public hospitals are both under the management of health authorities but are separate public institutes, thus patient information was often not shared during the referral [34].

PCAT scores reported in the two cities were similar to those reported in Taiwan where primary care providers do not play a gate-keeping role [15], but lower than those reported in Canada where primary care providers assumed the full role of gatekeepers based on long-term patient-doctor relationship [35]. Patients tend to have more effective communications with their doctors and report better health outcomes in countries where primary care providers act as gatekeepers [36].

This study found a strong association between income and quality of primary care in Hong Kong but not in Shanghai. Similar trends were identified in Hong Kong when including users of private primary care providers [37] and in countries with a large for-profit private sector in primary care [38, 39]. Quality of primary care tends to be equitable in better primary care oriented systems such as the UK [40]. In Shanghai, preventive care is financed by the government, while acute care is funded by health insurance schemes with a small percentage of out-of-pocket payments. The majority of Shanghai patients perceived acute care in CHCs as inexpensive and acceptable [41].

Shanghai has clearly defined the catchment area for CHCs, and then financed and staffed CHCs on per capita basis according to their population since 2006 [42]. Countries with a well-defined area and population for primary care providers tend to achieve better accessibility and continuity in primary care, because providers know better of their service population [6, 43]. Hong Kong does not set staffing levels for GOPCs [44]. The current staffing allocation of GOPCs are largely based on volume of attendances in 2003, while the majority of primary care is provided by private doctors whose practice are not monitored by the government. Patients in Hong Kong tend to visit a number of doctors in a single disease episode due to the lack of long-term doctor-patient relationship [44].

One major limitation is that the PCAT scores may not fully reflect the different health system factors in Hong Kong and Shanghai that may influence the quality of primary care. For example, regarding health delivery, over 70% of primary care in Hong Kong is provided by private doctors while 20% is by public GOPCs. In Shanghai, primary care is provided by public CHCs and hospitals. Bao and colleagues reported that 65% of Shanghai residents preferred CHCs while 21% visited big hospitals for primary care.[11] The different mix of primary care providers may influence patient perceived care quality as patient tends to compare their experiences with their peers. However, in both settings, the elderly and the poor are more likely to visit public primary care providers, so their experience is comparable between the two cities. Another influencing factor is health insurance. GOPCs in Hong Kong are fully funded by the public, while CHCs in Shanghai receive their revenues from the government and public health insurance schemes. The Health Insurance Bureau in Shanghai has direct influence on CHCs, such as limiting cost per prescription.[42]

Several other limitations should be considered. First, we conducted a telephone survey in Hong Kong but an on-site survey in Shanghai. Face-to-face interview tends to yield higher response rate than telephone interview. However, we achieved a relatively high response rate in the telephone survey. We did not conduct telephone survey in Shanghai because population-based household telephone dictionary is not available in Shanghai. Second, the nature of survey prevents studies of any causal relationship. Third, recall bias might be introduced but there is no reason to believe that the bias occurred systematically. Forth, we did not include patients in Shanghai who rely on public hospitals as their primary source of care because direct use of specialist care is unsustainable and discouraged by the government policies. Fifth, we did not test the reliability of the questionnaire between the two dialects of Chinese, though both the Cantonese and Mandarin versions of PCAT have been validated and contained the same questions. Last, we were not able to establish statistical linkages between health system factors, such as financing and organization, and quality of primary care. Future studies may explore this in order to provide stronger evidence.

## Conclusion

Our study suggested that higher quality of primary care was reported in Shanghai compared with Hong Kong. To improve primary care, Hong Kong needs to improve interpersonal continuity of care and involve more preventative care in GOPCs, while Shanghai needs to strengthen the capacity of CHCs towards fully functional health gatekeepers. The quality of public primary care tended to be more equitable in Shanghai where the CHCs have defined catchment areas and focus on preventative care, compared with the laissez faire public primary care system in Hong Kong.

## Supporting Information

S1 Appendix Survey questionnaire, the adult primary care assessment tool. (DOCX)
