# Supplementary material for: Comparing Quality of Public Primary Care between Hong Kong and Shanghai Using Validated Patient Assessment Tools
Source: PLoS One. 2015 Mar 31;10(3):e0121269. doi: 10.1371/journal.pone.0121269 (PMC4380428; doi:10.1371/journal.pone.0121269)
Supplement: S1 Appendix — (DOCX) [file pone.0121269.s001.docx]

**香港中文大學公共衛生及基層醫療學院**

**應用系統架構評估香港、深圳、昆明和上海的基層保健服務研究項目**

**基層醫療服務使用者之基層衛生保健使用評估**

**您好，请问是否方便和您做一个访谈**

**1 可以（继续至同意书）**

**2 不方便（询问另外方便的时间再打电话或者在面对面访谈中谢谢对方）**

**Is this a convenient time for you to talk with me?**

**1 Yes (Go to consent)**

**2 No (Ask for another time for call for telephone survey or just thanks for face to face interview)**

| 1. **與醫生聯繫的程度 （3条问题）**EXTENT OF AFFILIATION WITH A PLACE/DOCTOR | | |
| --- | --- | --- |
| A1. | Is there a doctor or place that you usually go if you are sick or need advice about your health?  當你生病或需要健康上的意見的時候，你是否有一位通常會見的醫生或者會求醫的地方以徵詢意見。  当你不舒服或有健康问题的时候，是否经常去某个社区卫生服务中心看病？ | |
|  | A 否 No |  |
|  | b 是 請提供求醫地方名字 Yes (Please give name and address.)： |  |
|  | What’s the place belong to: a. General Out-Patient Clinics under HA; b. Private clinics; c. Chinese Medicine Clinics (If not a,b,c, then thank you, hope to get you opinion next time.—Cut line) 請問該地方是 a醫院管理局普通科門診 b私家診所 c 中醫門診或診所 （如非a,b,c則感謝你的時間，希望下次有機會聆聽您的意見） |  |
| A2. | Is there a doctor or place that knows you best as a person?  是否有一位醫生或者你會求醫的地方很瞭解你？  是否有一个社区卫生服务机构很了解您的情况？ |  |
|  | a 否 No |  |
|  | b 是, 並與上述醫生/地方相同 Yes, same place as above |  |
|  | c 是, 但與上述醫生/地方不同 ，請提供求醫地方名字 Yes, different place (Please give name and address.)： |  |
| A3. | Is there a doctor or place that is most responsible for your health care?  是否有一位醫生或者會求醫的地方對你的健康負責？ |  |
|  | A 否 No |  |
|  | b 是, 並與 #A1及#A2 所述的醫生/地方相同Yes, same as #A1 & #A2 above |  |
|  | c 是, 但只與 #A1所述的醫生/地方相同Yes, same as #A1 only |  |
|  | d 是, 但只與 #A2所述的醫生/地方相同Yes, same as #A2 only |  |
|  | e 是, 但與#A1 及 #A2所述醫生/地方不同，請提供求醫地方名字Yes, different from #A1 & #A2 (Please give name and address.) ： |  |
|  | | |
| 若上述三題的答案都為 “否”，請提供你上次求診的地方：If you answered NO to all three questions, please write in the name of the *last* doctor or place you went to:  地方名字Name of doctor or place:： | | |
|  |  |  |
|  | | |

若上述三題答案相同， 請以該醫生/地方回答以下問題（請轉至下一頁）

If all three places are the same, please answer all the rest of the questions about this doctor or place. (Go to next page.)

若其中兩題答案相同，請以此兩題答案的醫生/地方回答以下問題（請轉至下一頁）

If any two of the places are the same, please answer all the rest of the questions about that doctor or place. (Go to next page.)

若上述三題的答案不同，請以A1答案提及的醫生/地方回答以下問題（請轉至下一頁）

If all three places are different, answer all the rest of the questions about the doctor or place in question A1. (Go to next page.)

若其中兩題回答為“否”，請以答案為“是”的醫生/地方回答以下問題（請轉至下一頁）

If you answered NO to two questions, answer all the rest of the questions about the doctor or place in the question you answered YES. (Go to next page.)

若上述三題的答案都為“否”，請寫下你上次求診地方的地址： 並以此地方回答以下問題（請轉至下一頁）

If you answered NO to all three questions, please write in the name of the *last* doctor or place you went to and answer the following question according to this place

| Name of doctor or place: |
| --- |

以下問題，我們會將該醫生/地方稱為“你的醫生”或者“該診所”

We will call this doctor or place your PCP in all the rest of the questions.

A8 About *how many times total* have you been there? _______ times

過去一年中，你總共去到該診所求診大概多少次？ _______ 次

A9 你於該诊所求診的時間有多長？How long have you been going there?

|  | 1. 少於6個月Less than 6 months | 2. 6個月至1年  Between 6 months and one year | 3. 1至2年  1 – 2 years | 4. 3-4年  3 – 4 years |
| --- | --- | --- | --- | --- |
|  | 5. 5年或以上5 or more years | 6. 多變的, 很難具體說明Too variable to specify | 9. 不肯定/已忘記Not sure/don’t remember |  |

| **B. 可達性　－　使用** （5Qs）FIRST CONTACT – UTILIZATION | | | | | |
| --- | --- | --- | --- | --- | --- |
| 請選擇一個最合適答案．Please check the **one** best answer. | 一定會  Definitely | 可能會  Probably | 可能不會  Probably not | 一定不會  Definitely not | 不肯定／已忘記  Not sure/don’t  remember |
| B1. When you need a regular general checkup, do you go to your PCP before going somewhere else?  當你需要一般身體檢查時，你會首先選擇見你的醫生嗎？  当你需要体检时，会首先来这里吗？ | 4 | 3 | 2 | 1 | 9 |
| B2. When you have a new health problem, do you go to your PCP before going somewhere else?  當你有一個新的健康問題時，你會首先選擇見你的醫生嗎？  当您觉得身体不舒服时，首先来这里看病的可能性为多大？ | 4 | 3 | 2 | 1 | 9 |
| B3. When you have to see a specialist, does your PCP have to approve or give you a referral?  當你需要專科治療時，你會需要你的醫生為你轉介嗎？  当你需要看专科医生是，会需要这里为你转诊吗？ | 4 | 3 | 2 | 1 | 9 |

BA1. How do you go to your PCP? 1. Walking 2. Driving/taking taxi 3. Bus 4 other: ______

您如何去見你的醫生？ 1. 行路 2. 開車或的士 3. 巴士 4. 其他:______

你如何到达这里？ 1.走路 2. 开车或的士 3.公交车 4自行车或电动车 5. 其他

BA2. How long it takes for you to get to your PCP? _________Minutes

你到達你的醫生處需要幾多分鐘? _________分鐘

您到这里路途________分钟

| **C. 可達性　－　可接近性 (4 Qs)** | | | | | |  |
| --- | --- | --- | --- | --- | --- | --- |
| 請選擇一個最合適答案．  Please check the **one** best answer. | 一定會  Definitely | 可能會  Probably | 可能不會  Probably not | 一定不會  Definitely not | 不肯定／已忘記  Not sure/don’t  remember | |
| C3. When your PCP is *open* and you get sick, would someone from there see you the same day?  當你生病而你的醫生又有應診，你會否當日見到醫生？  您来这里看病能当日见到医生吗？ | 4 | 3 | 2 | 1 | 9 | |
| C4. When your PCP is *open*, can you get advice quickly over the phone if you need it?  如果你的醫生有應診，你可以打電話快捷地得到建議  在营业时间，您可以打电话来这里咨询吗 | 4 | 3 | 2 | 1 | 9 | |
| C5. When your PCP is *closed*, is there a phone number you can call when you get sick?  當你生病而你的醫生又休息，你亦可以打電話联络到你的医生得到建議  在非营业时间，是否有一个电话号码您可以打来咨询吗？ | 4 | 3 | 2 | 1 | 9 | |
| C7. When your PCP is *closed* and you get sick *during the night*, would someone from there see you that night?  如果你需要，你的醫生在任何時候都可以為你診治，就算在夜晚或者其他休息時間？  在非营业时间，这里的医生是否可以为你诊治？ | 4 | 3 | 2 | 1 | 9 | |

| **D.可持續性** (4 Qs) ONGOING CARE | | | | | | | |  |
| --- | --- | --- | --- | --- | --- | --- | --- | --- |
| 請選擇一個最合適答案．Please check the **one** best answer. | 一定會  Definitely | 可能會  Probably | | 可能不會  Probably not | | 一定不會  Definitely not | 不肯定／已忘記  Not sure/don’t  remember | |
| D1. When you go to your PCP’s, are you taken care of by the *same* doctor or nurse each time?  當你到該診所求診時，你每次都會見同一個醫生或護士嗎？  您每次来这里，会不会每次都看同一位医生？ | 4 | | 3 | | 2 | 1 | 9 | |
| D4. If you have a question, can you call and talk to *the doctor or nurse who knows you best*?  如你有疑問，你可以打電話詢問最熟悉你的醫生或護士嗎?  您平时有什么健康问题是否可以和这里的医护人员交流？ | 4 | | 3 | | 2 | 1 | 9 | |
|  | 一定是  Definitely | | 可能是  Probably | | 可能不是  Probably not | 一定不是  Definitely not | 不肯定／忘記  Not sure/don’t  remember | |
| D7. Does your PCP know you very well as a *person*, rather than as someone with a medical problem?  你的醫生是否很瞭解你,而並不只當你是一個有病的人/病例  这里的医护人员是否不仅仅把您当做一位病人，还很了解您的其他情况？ | 4 | | 3 | | 2 | 1 | 9 | |
| D9. Does your PCP know what problems are most important to you?  你的醫生是否很瞭解對你來講最重要的問題是甚麼？  这里的医护人员是否知道您最重要的健康问题？ | 4 | | 3 | | 2 | 1 | 9 | |

| **E. 分科合作** (5 Qs) COORDINATION | | | | | |
| --- | --- | --- | --- | --- | --- |
| E2. Have you ever had a visit to any kind of specialist or special service?  你曾否接受專科醫生的診治或其他特別服務？  您有没有去过其他大医院或专科医院看病呢？  1. 有 Yes 2. 沒有 →跳至F1 No (**Skip to question F1**.)  3. 不肯定/已忘記 →跳至F1 Not sure/don’t remember (**Skip to question F1**.) | | | | | |
| 請選擇一個最合適答案．Please check the **one** best answer. | 一定會  Definitely | 可能會  Probably | 可能不會  Probably not | 一定不會  Definitely not | 不肯定/忘記Not sure/don’t  remember |
| E7. Did your PCP know you made these visits to the specialist or special service?  你的醫生會否知道你曾接受專科醫生診治或專科服務?  这里的医生有没有知道您去大医院或专科医院治疗？ | 4 | 3 | 2 | 1 | 9 |
| E8. Did your PCP discuss with you different places you could have gone to get help with that problem?  你的醫生有無同你商討你可到其他地方治療你的問題？  这里的医生是否会和您讨论其他地方的就医选择？ | 4 | 3 | 2 | 1 | 9 |
| E9. Did your PCP or someone working with your PCP help you make the appointment for that visit?  你的醫生或該診所職員有無過幫你預約該次治療？  当您需要转诊时，这里的医护人员会帮您联系大医院吗？ | 4 | 3 | 2 | 1 | 9 |
| E10. Did your PCP write down any information for the specialist about the reason for the visit?  你的醫生有無寫低你需要專科診治的原因給該專科醫生？  当您需要转诊时，这里的医生会在您的病历本上写下转诊的原因（或填写转诊单）吗？ | 4 | 3 | 2 | 1 | 9 |

| **F. 分科合作（資訊）**(3Qs) COORDINATION (INFORMATION SYSTEMS) | | | | | | | | | |
| --- | --- | --- | --- | --- | --- | --- | --- | --- | --- |
| 請選擇一個最合適答案．Please check the **one** best answer. | | 一定會  Definitely | | 可能會  Probably | | 可能不會  Probably not | 一定不會  Definitely not | | 不肯定/忘記Not sure/don’t  remember |
| F1. When you go to your PCP, do you bring any of your own medical records, such as shot records or reports of medical care you had in the past?  當你到該診所求診時，你會攜帶你個人的病歷紀錄，如以往的防疫注射或醫療報告嗎？  当您前往这里是，会携带病历本吗？ | 4 | | 3 | | 2 | | | 1 | 9 |
| F2. Could you look at your medical record if you wanted to?  如你要求，你能查看你個人的病歷紀錄  这里是否由您看病的所有记录？ | 4 | | 3 | | 2 | | | 1 | 9 |
| F3. When you go to your PCP, is your medical record always available?  當你到該診所求診時，你的病歷紀錄經常都已準備好  您每次来看病时，医生都会参考您以前的看病记录吗 | 4 | | 3 | | 2 | | | 1 | 9 |

| **G. 綜合性（可用服務）**(4 Qs) COMPREHENSIVENESS (SERVICES AVAILABLE) | | | | | |
| --- | --- | --- | --- | --- | --- |
| Following is a list of services that you or your family might need at some time. For each one, please indicate whether it is available at your PCP’s office.  據你所知，你的醫生會提供以下的服務嗎？  以下是一些常用的健康服务，据您所知，这里会提供以下服务吗？请选择一个最合适的答案。不一定是您个人的经验，只是就您了解的情况回答 | 一定  會  Definitely | 可能  會  Probably | 可能不會  Probably not | 一定不會  Definitely not | 不肯定／忘記  Not sure/don’t  remember |
| G2. Immunizations (shots) 防疫注射  打预防针（疫苗注射接种） | 4 | 3 | 2 | 1 | 9 |
| G6. Family planning or birth control methods  家庭計劃或避孕  计划生育技术指导（或避孕指导） | 4 | 3 | 2 | 1 | 9 |
| G8. Counseling for mental health problems  精神健康問題咨詢或心理健康輔導 | 4 | 3 | 2 | 1 | 9 |
| G10. Sewing up a cut that needs stitches  傷口縫合  外科医疗（如上课缝合、包扎换药） | 4 | 3 | 2 | 1 | 9 |

| **H. 綜合性（服務提供）**（5 Qs）COMPREHENSIVENESS (SERVICES PROVIDED) | | | | | |
| --- | --- | --- | --- | --- | --- |
| In visits to your PCP, are any of the following subjects discussed with you?  以下問題涉及你有時可得到的醫療服務．  當你求診時，你的醫生曾否與你談及以下問題？有些問題可能比較少見, 請選擇一個最合適答案．  当您来这里是，医护人员和您提及一下健康问题多的频率如何？请就您个人的经验来回答 | 一定  有  definitely | 可能  有  Probably | 可能無 probably not | 一定無Definitely not | 不肯定／忘記  Not sure/don’t  remember |
| H1. Advice about healthy foods and unhealthy foods or getting enough sleep  健康與不健康食物或充足睡眠之意見  饮食指导（限盐限油），或充足睡眠 | 4 | 3 | 2 | 1 | 9 |
| H2. Home safety, like getting and checking smoke detectors and storing medicines safely  家居安全，如防火、安全儲存藥物  居家安全（比如防范火灾、安全存放药物等） | 4 | 3 | 2 | 1 | 9 |
| H4. Ways to handle family conflicts that may arise from time to time  處理家庭糾紛的方法 | 4 | 3 | 2 | 1 | 9 |
| H5. Advice about appropriate exercise for you  有關適合你的運動的意見  建议您做适合的运动 | 4 | 3 | 2 | 1 | 9 |
| H7. Checking on and discussing the medications you are taking  檢查及討論你正在服用的藥物  询问您服药的情况 | 4 | 3 | 2 | 1 | 9 |

| **I. 以病人及家庭為中心** (3 Qs) FAMILY-CENTEREDNESS | | | | | |
| --- | --- | --- | --- | --- | --- |
| These next questions are about the relationship of your health care providers with your family. Please check the **one** best answer.  這部份的問題是關於你的家人與你的醫生的關係．請選擇一個最合適答案． | 一定會/一定有  definitely | 可能會/可能有Probably | 可能不會  /可能無 Probably not | 一定不會/一定無Definitely not | 不肯定／已忘記  Not sure/don’t  remember |
| I1. Does your PCP ask you about *your* ideas and opinions when planning treatment and care for you or a family member?  在安排你或你家人的治療計劃時，你的醫生會徵詢你的意見嗎  这里的医生在为您制定治疗方案时，会征求您的意见吗？ | 4 | 3 | 2 | 1 | 9 |
| I2. Has your PCP asked about illnesses or problems that might run in your family?  你的醫生有無詢問過你的家族遺傳疾病或問題  这里的医护人员会询问可能在您的家族遗传病史吗 | 4 | 3 | 2 | 1 | 9 |
| I3. Would your PCP meet with members of your family if you thought it would be helpful?  如你認為有需要時，你的醫生會與你的家人會面嗎  如您需要，这里的医护人员会与您的家人一起讨论对您的治疗吗 | 4 | 3 | 2 | 1 | 9 |

| **J. 社區定向** (3 Qs) COMMUNITY ORIENTATION | | | | | |  |
| --- | --- | --- | --- | --- | --- | --- |
| Please check the **one** best answer.  請選擇一個最合適答案． | 一定會  Definitely | 可能會  Probably | 可能不會  Probably not | 一定不會  Definitely not | 不肯定/忘記Not sure/don’t  remember | |
| J1. Does anyone at your PCP’s office ever make home visits?  該診所會有職員做家訪嗎  这里的医护人员有没有去过您家中进行家访服务吗 | 4 | 3 | 2 | 1 | 9 | |
| J2. Does your PCP know about the important health problems of your neighborhood?  你的醫生會否知道你的居住地區有哪些重要的健康問題  这里的医护人员和您谈过需防范那些社区内的流行病吗 | 4 | 3 | 2 | 1 | 9 | |
| J3. Does your PCP get opinions and ideas from people that will help to provide better health care?  你的醫生會徵詢其他可以提供更佳醫護服務的人的意見  这里的医护人员会不会和社区或街道的工作人员联系来提供更好的服务呢？ | 4 | 3 | 2 | 1 | 9 | |
| **K.與不同文化的溝通** (2 Qs) CULTURALLY COMPETENT | | | | | | |
| 請選擇一個最合適答案．Please check the **one** best answer. | 一定會  Definitely | 可能會  Probably | 可能不會  Probably not | 一定不會  Definitely not | 不肯定/忘記Not sure/don’t  remember | |
| K1. Would you recommend your PCP to a friend or relative?  你會推薦你的醫生給你的朋友或者親戚嗎？  您会推荐您的亲戚朋友来这里或找某位医生就诊吗 | 4 | 3 | 2 | 1 | 9 | |
| KA1. Would you recommend your PCP to your friends or relatives for their TCM treatment?  你會推薦你的醫生給你的朋友或者親戚接受中醫服務嗎？  您会推荐您的亲戚朋友来这里接受中医服务吗？ | 4 | 3 | 2 | 1 | 9 | |

| **L.保險問題** (7 Qs) INSURANCE QUESTIONS | | | |
| --- | --- | --- | --- |
| **L1** How much of the past 12 months were you covered by *any* type of health insurance?  **在过去一年中，您有多少时间是有本地医疗保险的？**  4 未曾参加保险never 3小于六个月 less than 6 months 2 六个月至一年 6 month to 1 year 1 全年 all year 9不肯定 not sure | | | |
| During the last 12 months, was any of your health care paid through the following ways:  過去一年中，你有否通過以下方式支付你的醫療服務?  过去一年里，您是如何支付你哪的医药费的？ | 有 Yes | 無 No | 不肯定／忘記Not sure/don’t  remember |
| L3. Private Insurance Company  私人醫療保險公司  通过私人保险公司支付 | 1 | 2 | 9 |
| L5. Some governmental health department clinic  公立醫院及診所費用減免機制  社会医保支付 | 1 | 2 | 9 |
| LA1. Insurance provided by employer  雇主提供的醫療保險福利 | 1 | 2 | 9 |
| L6. Personal income (cash, check, credit card)  個人收入（現金、支票或信用卡）  现金支付 | 1 | 2 | 9 |

L8. 其他 (請註明)other, please write down

LA2. Do you think the amount you paid for medical care is acceptable?

在過去一年,你認為你付於醫療服務的金額是否可以接受?

您认为您在这里看病支付的医疗服务费用是否能接受

|  | 4. Acceptable  可以接受  能接受 | 3. Fairly acceptable  一般  还可以 | 2. hard to accept很難接受  不能接受 | 1. Not affordable無力負擔   无法负担 | 9. don’t know/not sure  不肯定  不肯定 |
| --- | --- | --- | --- | --- | --- |

| **M. 自我健康評估** (2 Qs) HEALTH ASSESSMENT |
| --- |
| M1. Would you say your health status is:  你覺得你自己的健康是：  您觉得您的健康状况是  1. 極佳 Excellent 2. 很好 Very good 3. 好Good 4. 尚可 Fair 5. 差Poor  M2. Do you have any physical, mental, or emotional problem that has lasted or is likely to last longer than one year? 你有沒有任何已經或可能會持續超過一年的身體或情緒問題？  您是不是有任何已确诊的慢性病  1. 有 Yes 2. 沒有 No 9. 不肯定/已忘記Not sure/don’t remember |
